# Sampling and Mass Detection of a Countable Number of Microparticles Using on-Cantilever Imprinting

**DOI:** 10.3390/s20092508

**Published:** 2020-04-28

**Authors:** Wilson Ombati Nyang’au, Andi Setiono, Angelika Schmidt, Harald Bosse, Erwin Peiner

**Affiliations:** 1 Institute of Semiconductor Technology (IHT) and Laboratory for Emerging Nanometrology (LENA), Technische Universität Braunschweig, D38106 Braunschweig, Germany; a.setiono@tu-braunschweig.de (A.S.); angelika.schmidt@tu-braunschweig.de (A.S.); e.peiner@tu-bs.de (E.P.); 2 Department of Metrology, Kenya Bureau of Standards (KEBS), 00200 Nairobi, Kenya; 3 Research Center for Physics, Indonesian Institute of Sciences (LIPI), Kawasan Puspiptek Serpong, Tangerang Selatan 15314, Indonesia; 4 Precision Engineering Division, Physikalisch-Technische Bundesanstalt (PTB), 38116 Braunschweig, Germany; Harald.Bosse@ptb.de

**Keywords:** piezoresistive microcantilever mass sensor, resonant frequency, dispensing tip, droplet, particle sampling, adsorption, PMMA, magnetic polystyrene particles

## Abstract

Liquid-borne particles sampling and cantilever-based mass detection are widely applied in many industrial and scientific fields e.g., in the detection of physical, chemical, and biological particles, and disease diagnostics, etc. Microscopic analysis of particles-adsorbed cantilever-samples can provide a good basis for measurement comparison. However, when a particles-laden droplet on a solid surface is vaporized, a cluster-ring deposit is often yielded which makes particles counting difficult or impractical. Nevertheless, in this study, we present an approach, i.e., on-cantilever particles imprinting, which effectively defies such odds to sample and deposit countable single particles on a sensing surface. Initially, we designed and fabricated a triangular microcantilever sensor whose mass *m*_0_, total beam-length *L*, and clamped-end beam-width *w* are equivalent to that of a rectangular/normal cantilever but with a higher resonant frequency (271 kHz), enhanced sensitivity (0.13 Hz/pg), and quality factor (~3000). To imprint particles on these cantilever sensors, various calibrated stainless steel dispensing tips were utilized to pioneer this study by dipping and retracting each tip from a small particle-laden droplet (resting on a hydrophobic *n*-type silicon substrate), followed by tip-sensor-contact (at a target point on the sensing area) to detach the solution (from the tip) and adsorb the particles, and ultimately determine the particles mass concentration. Upon imprinting/adsorbing the particles on the sensor, resonant frequency response measurements were made to determine the mass (or number of particles). A minimum detectable mass of ~0.05 pg was demonstrated. To further validate and compare such results, cantilever samples (containing adsorbed particles) were imaged by scanning electron microscopy (SEM) to determine the number of particles through counting (from which, the lowest count of about 11 magnetic polystyrene particles was obtained). The practicality of particle counting was essentially due to monolayer particle arrangement on the sensing surface. Moreover, in this work, the main measurement process influences are also explicitly examined.

## 1. Introduction

The need and demand for a cost-effective and reliable fluid-based particles sampling and counting technique is of an inestimable significance, e.g., in biomedicine, to detect physical/chemical and biological particles, and diagnosis of diseases [1], etc. Conveyance and gravimetric detection of these particles with suspended microchannel resonators [2,3] has recently been achieved with negligible damping and high mass sensitivity. The sensors (e.g., microcantilevers) can also, for instance, be dipped into a solution containing particles or analytes of interest such as the feline coronavirus to adsorb and detect them [4,5,6]; but, low mechanical quality factor *Q* and randomized particles adsorption are the inevitable outcomes. Alternatively, liquid-borne media can most conveniently be transferred onto a sensing surface through droplet dispensing coupled with solvent evaporation. Nonetheless, a ring-cluster of particles (also called coffee-ring effect) is often observed at the edges of a dried liquid droplet [7,8,9,10]. This is a typical phenomenon that is manifested, for instance, after the evaporation of impure water droplets on a solid surface, deposition of DNA/RNA microarrays with functional and particle coatings [11], disease diagnostics and drug discovery [12], lithography patterning [13], particle and biomolecule separation and concentration [11]. 

The coffee-ring phenomenon is majorly caused by the pinning of a contact line of the drop edges to the substrate, and the radial outward-flow from the center (of the droplet) of carrier liquid during evaporation, which eventually transports the suspended particles to the rim [14]. Moreover, the particles should adhere to the substrate surface and the evaporation rate be high near the edge of the droplet. Consequently, the solvent that is lost to the ambient atmosphere (through evaporation at the rim of the droplet) is primarily compensated by the fluid flow (accompanied with the solutes/particles) from the center of the droplet. 

**Figure 1:**
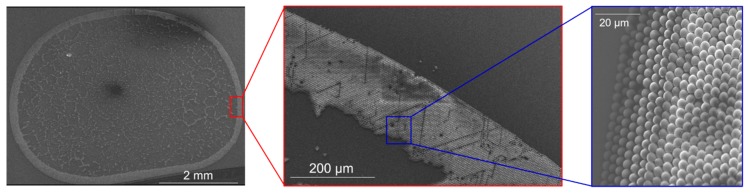
Typical SEM image of a cluster-ring deposit of polymethylmethacrylate (PMMA) particles arising from droplet dispensing on an *n*-type (100) silicon-substrate surface.

The particle ring deposits have, however, been eliminated or suppressed by various techniques. For instance, Yuinker et al. (2011) used ellipsoidal-shaped or a mixture of both spherical and a small number of ellipsoidal suspended particles [15] to suppress the cluster-ring effect. Elsewhere, the ring phenomenon has been managed and suppressed by controlling and optimizing of drop temperature [16], using surfactants [17], and tuning the particle concentration and droplet size [18], etc. It should be noted, however, that in cases where determination of particle concentration (or number of particles) is necessary, the cluster-ring deposits (see Figure 1) make particles counting extremely difficult or even impractical. The latter is quite explicit particularly if the adsorbed particles form non-uniform multilayers on the solid surface. By tuning the particle concentration, conventional liquid dispensing [19,20] can be utilized to deposit and realize a relatively small particle concentration [18]. This is, however, a pressure-driven process, and the dispensing tips are often inevitably clogged [20]. With dip-pen nanolithography [21,22], an atomic force microscope (AFM) tip (used as a pen) is dipped into a desired molecular ink; and then the sampled ink (coated on the apex of the atomically sharp tip) is transferred directly onto the substrate (from the tip/meniscus to the meniscus/surface interface). But this is a serial process characterized with low throughput. Moreover, limited substrates and inks can be used with this method. Additionally, the expensive and fragile micro/nano-sized AFM tips deployed in this scanning-probe-based direct-writing method limits the versatility of the technique. Similarly, using a polymer stamp, i.e., poly(dimethylsiloxane) (PDMS), with a predesigned pattern, micro-contact-based printing [23] can be applied to pattern self-assembled monolayers (SAMs) and deliver numerable particles onto substrate surfaces. This approach is however difficult to integrate with resonant mass sensors.

Consequently, an easy-to-implement practical approach for assembling a monolayer or uniformly multi-layered particles on a sensing surface is of desirable interest. In this study, a particle-imprint method is presented as a flexible and versatile approach for delivering small countable amounts of particle samples on solid surfaces. Unlike droplet dispensing, this method does not require dispensing air pressure nor complicated equipment. Thus, making it a cost-efficient alternative technique for sampling and depositing particles on sensing surfaces.

The particle-imprint method (in this work) involves dipping a dispensing tip into an arbitrary sized particles-laden droplet followed by substrate/sensor contact to deposit the particles. With this method, a specified tip can be dipped and retracted from a small droplet containing assorted monodispersed microparticles (µPs). Afterwards, the particles-laden tip immediately contacts the cantilever sensing area for a defined time duration (to deposit the particles). During this tip/sensor contact period, we have shown that some particle solution detaches from the tip and adheres onto a hydrophilic sensing surface (i.e., silicon bulk substrates and microcantilevers). In congruence with our initial hypothesis, monolayer particles arrangement on these sensors and substrates has also been realized.

In the subsequent sections, we therefore present details on how assorted particle samples, i.e., PMMA and magnetic polystyrene particles, were localized and deposited onto in-house fabricated microcantilever sensors utilizing our present approach and setup. PMMA particles (in a functionalized state, i.e., if the particle surface is bound with surfactant molecules) are most widely used as biomedical materials due to their biocompatibility. Recently, these particles have increasingly been applied as drug carriers (e.g., antibiotics), fillers for cosmetic and dental surgery, and for vaccine formulation [24] and colon cancer treatment [25]. Moreover, their application in colloidal lithography has recently been demonstrated [26]. Similarly, functionalized magnetic polystyrene particles have found manifold applications in various fields. Of interest is their use in separating biomolecules (e.g., antibodies, proteins, and nucleic acids etc.) [27], and separating and sorting of cells [28]**.** They are also utilized as tracers for magnetic particle imaging (MPI) [29], agents for diagnostics and targeted destruction of cancer tumors through local delivery of heat (hyperthermia), and for image contrast enhancement of diseased tissues [30] and targeted drug delivery [31]. In addition, they have notably been used in environmental pollution mitigation to remove oil from waste water [32]. In our study though, we have primarily utilized unfunctionalized forms of PMMA and magnetic polystyrene particles (as discussed in the subsequent section) and determined their mass concentration on the sensing surface. Further work to simultaneously determine the magnetic moment from the mass of magnetic polystyrene particles, utilizing a (modified) measurement setup, is intended. This will help to characterize and effectively render the use of these particles in magnetic resonance imaging (MRI) and MPI applications. In the current study, the particles samples (deposited by particle-imprint method) and adsorbed on in-house fabricated microcantilever mass sensors were quantified based on in-plane resonant frequency *f*_0_ response measurements and vividly compared with particle counts from scanning electron microscope (SEM) images.

## 2. Materials and Sensor Fabrication

### 2.1. Particle Samples

In this study, we used magnetic polystyrene particles (micromer®-M, hereafter denoted as MPS) from micromod Partikeltechnologie GmbH, Rostock, Germany; and polymethylmethacrylate (PMMA) from Sigma-Aldrich Inc., St. Louis, Missouri, USA. The nominal particle diameters (assuming spherical shape) and densities were about 2 μm and 1.1 gcm^−3^ (MPS µPs); 2 μm and 1.18 gcm^−3^ (PMMA µPs). Experimental samples were prepared by tuning the particle concentration by diluting the original solution with deionized water to realize various concentration levels ranging from approximately 0.01 mg/mL to 2 mg/mL.

### 2.2. Calibration of Dispensing Tips

**Figure 2:**
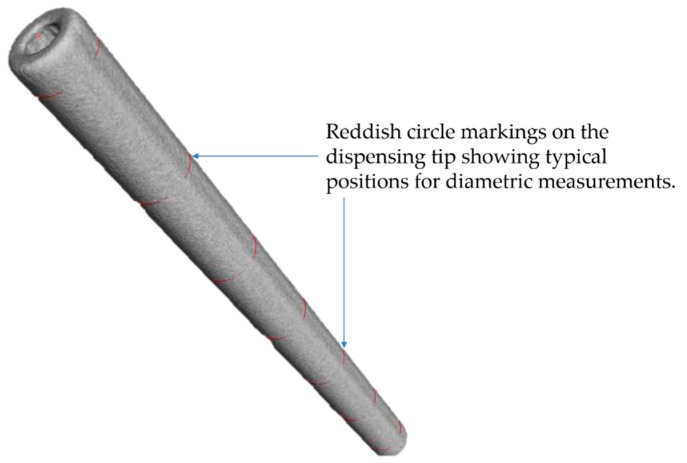
X-ray computed tomography (xCT) image showing a 3D rendering of the surface of capillary of a stainless-steel dispensing tip.

Prior to use, our stainless-steel dispensing tips were calibrated (at Physikalisch-Technische Bundesanstalt (PTB)) to determine their geometrical dimensions and shapes. This was performed using X-ray computed tomography (xCT) and optical reference measurements utilizing a coordinate measuring machine (CMM). The inner and outer diameters of the stainless-steel tip (from Nordson EFD Inc., East Providence, RI, USA), shown in Figure 2, were about 0.117 mm ± 10 µm and 0.236 mm ± 1 µm, respectively. The tips were cylindrical; and all the diametric measurements were taken at intervals of about 0.5 mm by fitting a circle to the determined surface. The assigned uncertainty values were computed with about 95 % confidence interval (*k* = 2). The main uncertainty factors considered included the repeatability of the measured diameters and the maximum permissible sphere distance error (for inner diameter) and maximum length measurement error (for outer diameter).

### 2.3. Cantilever Sensor Design and Fabrication

**Figure 3:**
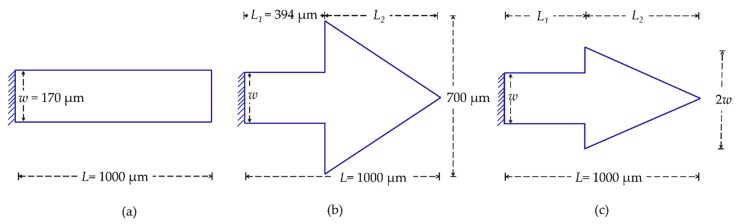
Schematic designs of (**a**) Rectangular (regular) cantilever (RCant1), and triangular cantilevers (**b**) first type (TCant1) and (**c**) second type (TCant2). The shape of the triangular free-end of TCant1 is equilateral (with the base *b* = 700 µm) whereas that of TCant2 is isosceles (with *b* = 2*w* = 340 µm).

In Table 1, we show the cantilever geometric dimensions and the simulated characteristics by finite-element modeling (FEM) using Comsol Multiphysics 4.4b. The free-end configurations of these microcantilevers (as depicted in Figure 3) were either rectangular or triangular, and the thickness *t* of all the sensors are essentially fixed (i.e., *t* = 15 µm). The triangular free-end of first type of triangular cantilever (TCant1, cf: Figure 3b) is equilateral-shaped (with sides = 700 µm, and length *L*_2_ = 606 µm), and it is positioned at *L*_1_ = 394 µm from the fixed-end of the cantilever (with beam width *w* = 170 µm). The length of the rectangular segment *L*_1_ = 394 µm ≈ 2/5*L*, where *L* is the total cantilever length of TCant1 i.e., *L*_1_ + *L*_2_; which corresponds to the length *L* = 1000 µm of the regular/rectangular cantilever (RCant1), as depicted in Figure 3a. The two sensors have different cantilever masses, i.e., *m*_0_ = 9.76 µg (TCant1) and *m*_0_ = 5.94 µg (RCant1). Similarly, their flexural fundamental resonant frequencies (and sensitivities) also differ significantly with *f*_0_ ≈ 185.0 kHz (→ ~ 0.04 Hz/pg) and *f*_0_ ≈ 220 kHz (→ ~ 0.07 Hz/pg) corresponding to TCant1 and RCant1 sensors, respectively. In the current study, we have designed a second type of triangular cantilever (TCant2, cf: Figure 3c) with an isosceles triangular-free-end whose base *b* (just like TCant1 sensor) is positioned at *L*_1_ = 394 µm (from fixed-end) but its magnitude is twice the clamping beam width (i.e., *b* = 2*w*). The total cantilever length *L* (i.e., from the fixed-end to apex/free-end) for TCant2 sensor was nonetheless the same as TCant1 and RCant1 sensors (i.e., *L* = 1000 µm). Additionally, by tuning the base-width *b* of the triangular-free end of TCant2 sensor (to *b* = 2*w*), the cantilever mass (*m*_0_ = 5.94 µg) is rendered equivalent to the mass of the regular cantilever (RCant1) sensor (having same total length *L* and fixed-beam width *w*). Although RCant1 and TCant2 sensors have the same *L* and *m*_0_, a larger resonant frequency and mechanical quality factor was, however, observed for TCant2 (*f*_0_ ≈ 271 kHz, *Q* ~ 3000 ± 150) than RCant1 (*f*_0_ ≈ 220 kHz, *Q* ~ 2000 ± 200), as shown in Table 1. This is supposedly due to their geometrical (shape) differences. Moreover, the isosceles (triangular fee-end) in TCant2 could possibly cause little damping and lesser effective cantilever mass compared to the rectangular counterpart. Nevertheless, further work is required to unravel the factors behind the observed differences. For cantilevers of equal length and similar free-end geometries but different triangular base-widths (→ different *m*_0_ values) i.e., TCant2 and TCant1 sensors, their fundamental in-plane resonant frequencies, mechanical quality factors, and sensitivities also differ accordingly (as shown in Table 1). TCant1 cantilever (*m*_0_ = 9.76 µg) yields a smaller *f*_0_ ≈ 185 kHz (→ ~ 0.04 Hz/pg) compared to *f*_0_ ≈ 271 kHz (→ ~ 0.09 Hz/pg) from TCant2 (*m*_0_ = 5.94 µg). Moreover, the latter offers an enhanced mass sensitivity and improved quality factor. It should be noted that all our sensors were designed to be excited in the in-plane bending mode of vibration. The main material parameters used in cantilever FEM simulations in Comsol Multiphysics were density (2.33 gcm^−3^), Young’s modulus (170 GPa) and Poisson ratio (0.28) for silicon, and volume (i.e., geometrical dimensions of the sensor). All our cantilevers are piezoresistive and work in a dynamic mode.

To manufacture these cantilevers, *n*-type (100) silicon wafers (Siegert Wafer GmbH, Aachen, Germany) were utilized as base material through a bulk-micromachining fabrication process [33,34]. Initially, these wafers were diced from the silicon wafers into ~ 30 × 30 mm^2^ substrates. Prior to use, the bulk silicon substrates were thoroughly cleaned to remove from their surfaces any organic contaminants (e.g., dust particles, lubricants, grease, silica gel, etc.), ionic contaminants (mostly from inorganic compounds), and silicon dust or metallic debris, etc. To clean them, each substrate was boiled (for ~5 min) in a 1:1 oxidant mixture solution of sulfuric acid (H_2_SO_4_, 96 %) and hydrogen peroxide (H_2_O_2_, 30 %) contained in a quartz glass beaker. The substrate was then immersed in a water bath for about 5 min before rinsing it with deionized water and blow drying with nitrogen gas. Such a cleaning process is oxidative; and it therefore yields a hydrophilic surface.

The main cantilever fabrication steps comprised of:

- Substrate preparation (i.e., pre-cleaning),
- Thermal oxidation,
- Photolithography,
- Dopant diffusions: *n*^+^ and *p* (1100 °C); *p*^+^ (1200 °C),
- Contact holes formation,
- Metallization: Cr: 300 Å / Au: 3000 Å,
- Membrane formation, in which the backside was etched in a cryogenic inductive-coupled plasma reactive ion etching (Cryo-ICP-RIE) process using SF_6_/O_2_,
- Structuring and free-release of cantilever through a second Cryo-ICP-RIE process (SF_6_/O_2_).

Basically, each pre-cleaned bulk silicon substrate (~30 × 30 mm^2^) was initially thermally oxidized (in a furnace at a temperature *T* ≈ 1100 °C for about 100 min). Subsequently, the oxidized sample was cleaned by sonication (using acetone as cleaning agent), then mounted and spin-coated with a positive photoresist (AZ 5214, Shipley) prior to patterning (by photolithography using MJB4 mask aligner from SÜSS MicroTec, Garching, Germany). Patterning was essentially useful in defining various microstructural features of interest ranging from *n*^+^, *p*, and *p*^+^ doping sites, contact holes, metallization, membrane, and the cantilever, respectively. Each of these features was realized using a specific mask design.

**Figure 4:**
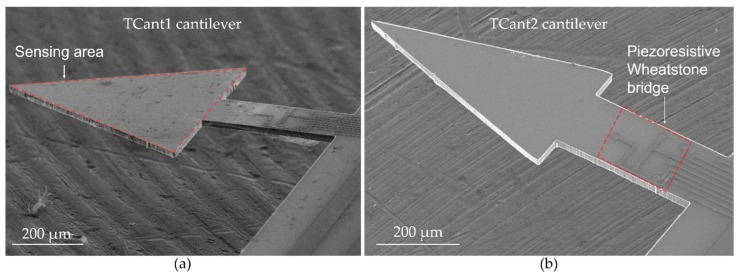
SEM images of silicon-based piezoresistive cantilever sensors after fabrication and basic cleaning processes, with (**a**,**b**) depicting TCant1 and TCant2 triangular cantilever sensors.

In patterning the metal-line connections, for instance, metallization was done by first depositing a 30-nm-chromium layer (which serves as an adhesive layer) and secondly, 300-nm-gold layer through an electron-beam deposition process. Subsequently, a lift-off process (in acetone) was carefully undertaken to remove the photoresist from the samples. Since this is a critical step in the fabrication process, a thorough microscopic inspection of the metallized samples was performed to assess the continuity of the connection lines. Hereafter, the sample was thermally oxidized and patterned for backside etching to define the membrane or thickness of the cantilever. Therefore, Cryo-ICP-RIE etching was carried out at a temperature of approximately −80 °C for approximately 56 min. Lastly, the cantilevers were patterned and freely released through a cryogenic etching process (at −95 C for about 15 min). This was the stage at which the desired free-end cantilever configuration (i.e., rectangular, or triangular) was realized, as depicted in Figure 4.

Usually, after fabrication, the cantilever surface was still covered with the photoresist. To remove it, the sensor was soaked and cleaned in acetone and thoroughly water-rinsed before drying in a gentle stream of nitrogen gas. Alternatively, oxygen plasma cleaning was performed. Both cleaning methods can reliably be used to clear the photoresist from the sensors. 

## 3. Particle-Imprinting Process 

### 3.1. Particle Sampling and Tip Coating

**Figure 5:**
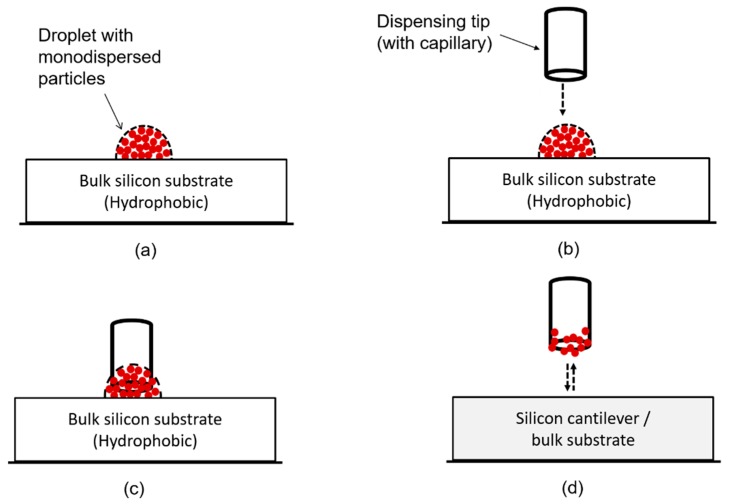
Schematic illustration of the particle sampling and deposition by particle-imprinting process. (**a**) Arbitrary sized droplet containing monodispersed particles on a hydrophobic silicon surface. (**b**) The dispensing stainless-steel tip is positioned above the droplet and then moved (using a 3D micro-positioning system) into the droplet. (**c**) The tip is dipped into the particles-laden droplet to coat it with the particles solution. (**d**) After retraction from the droplet, the particles-coated tip is moved onto the sensing surface to contact and deposit the particles thereon before it is retracted therefrom.

In Figure 5, we schematically illustrate the sampling and depositing of particles onto a silicon bulk substrate or cantilever-based sensor. Basically, two main steps were involved:

Firstly, a particle solution was initially prepared by homogenously mixing (by sonication) the selected monodispersed particle solution. Then, an arbitrary-sized small drop (see Figure 5a) was deposited on a pre-cleaned hydrophobic silicon surface (~10 ×10 mm^2^) under ambient conditions. In this case, the small droplet served as a particle reservoir. The particles, which are suspended in a fluid medium e.g. in a droplet, exhibit a random motion.

A dispensing stainless-steel tip (D-tip) was then moved (Figure 5b) and dipped into the small droplet (Figure 5c). Consequently, as illustrated in Figure 5d, the tip surface (apex) is coated (or inked) with the particles, i.e., a thin (liquid) film would form or adhere on the tip surface upon retraction from the droplet. The particle solution adheres on the surface of the tip courtesy of capillary action.

**Figure 6:**
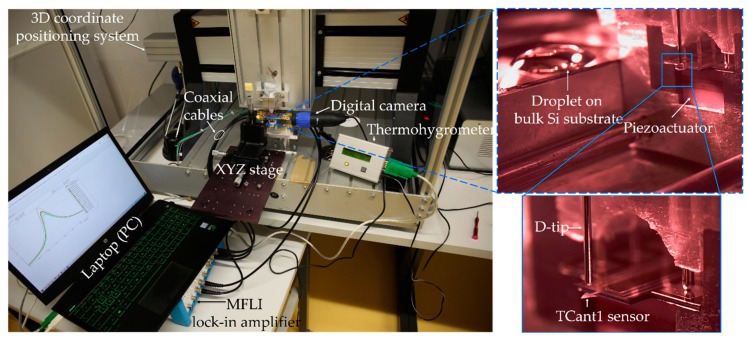
Optical image of the typical experimental setup utilized for tip-sensor contact process (particle deposition) and resonant frequency measurements (particles mass determination).

It should be noted that the tip apex and the sensing surface were carefully aligned in the same horizontal plane. Therefore, initially, a contact between the D-tip apex (without particle solution) and the surface was established, tested, and optically inspected using an USB camera (Mz-902, Oowl Tech Ltd., Hong Kong, China). The point of contact (i.e., *z*_o_) was carefully noted and the tip was then moved (by means of a micro-positioning system as depicted in Figure 6) into the particle reservoir (i.e., to point *z*_0_ in the drop) for particle coating (for a defined time period). 

### 3.2. Particle Transport Process 

The particle-coated tip was moved from the droplet to the target point (i.e., on the bulk silicon substrate or cantilever mass sensor surface) utilizing the micro-positioning system. Prior to this, the target surface e.g., the cantilever sensor (as depicted in Figure 6) was first mounted on a sampler (fixed on a workbench) followed by tip-alignment. The latter was performed by positioning the D-tip vertically above the cantilever at a defined point along the symmetry axis. The tip-on-sensor alignment process was optically aided by a camera (and was often repeated whenever the D-tip would be exchanged). The camera, in this case, helped to visualize the tip while moving and adjusting the tip apex as close as possible onto the sensor surface. This was necessary to mitigate and reduce the risk and possibility of breaking the fragile silicon cantilever sensor and therefore minimize the inaccuracies associated with tip misalignment which otherwise leads to an off-centered particle deposition/adsorption. Nonetheless, we estimated the minimum achievable tip-on-sensor position alignment accuracy to be about 10 µm. Taking due considerations, therefore, the coated tip was retracted from the droplet (to point *z*_1_), then moved laterally (along the y-axis), and moved vertically downwards (to point *z*_0_) to contact and imprint the particles on the sensing surface.

### 3.3. Particle Imprinting and Adhesion

The next step involved the transfer of the particles solution from the tip to the target (sensing) surface by mechanical contact, as depicted in Figure 5d. In this case, the particle-coated tip (apex) would contact the surface for a defined time duration, hereafter referred to as contact time *t_c_*. It is worth noting that tip-droplet dipping time corresponded to the tip-sensor contact time. During tip-sensing surface contact, the liquid is detached (from the tip) and attached onto the target surface. In case of a cantilever sensor, slight deflection is observed during tip contact; hence, avoiding the risk of breaking the sensor.

**Figure 7:**
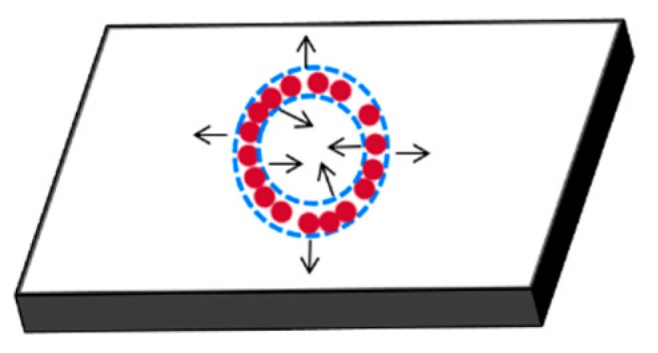
Schematic illustration of a particle-imprinted substrate sample. The inner radial liquid–air interface initiates inwards flow (in addition to outwards flow) to suppress the ring-clustering phenomenon.

After tip-sensor contact, the particles (along with the carrier fluid i.e., water) move radially outwards and inwards as depicted in Figure 7. During this process, particles arrange themselves on the surface with the solvent flow; and adsorption happens as a result of water evaporation and the inter-particle forces owing to surface tension. 

The amount of tip-adherent particles solution transferable to the target surface was mainly influenced by changing the wettability of the substrate/sensing surfaces, size of the dispensing tip, and tip-sensor contact time *t*_c_, using different types of particle solutions and particle concentration levels. Based on these dynamics, we present (in the next section) the typical outcomes of particle adsorption and arrangements on assorted bulk silicon substrates.

### 3.4. Particles Adsorption on Bulk Silicon Surfaces

Here, we investigate the influence of wettability of substrate surfaces on particle adsorption. Hydrophilic bulk silicon surfaces were basically realized after general cleaning of the substrates (i.e., boiling in a 1:1 oxidant mixture solution of H_2_SO_4_ (96%) and H_2_O_2_ (30%), as discussed in Section 2.3). Nevertheless, for enhanced hydrophilicity, these samples were further treated with O_2_ plasma (for ~30 s). For hydrophobic silicon substrates, the cleaned samples were dipped in a buffered 6 % to 7 % HF solution for about 10 s.

**Figure 8:**
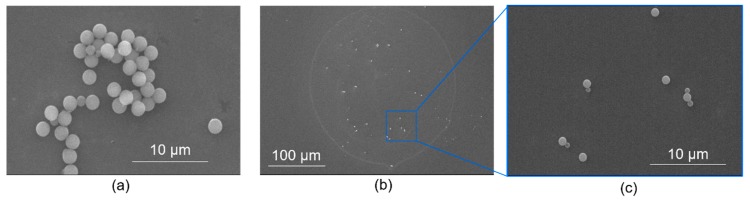
SEM images of particle-imprinted bulk silicon samples. (**a**) Partial segment of spherically shaped PMMA particles adsorbed on hydrophilic surfaces, (**b**,**c**) respectively depict a whole and magnified partial segment of PMMA particles adsorbed on a hydrophilic silicon surface. The particle-imprinting process was accomplished using a stainless-steel dispensing tip of inner/outer diameter ≈ 0.117/0.236 mm with a contact time of about 1 s.

In Figure 8, we show different substrate samples and their wettability conditions (i.e., with hydrophobic and hydrophilic surface treatments) and the particles-imprinting outcomes. In this case, we used a stainless-steel tip with inner and outer diameters of about 0.117 mm and 0.236 mm, respectively, and a contact time of ~1 s. In both surface wettability conditions, all the samples were evidently adsorbed with the particles. Nevertheless, for hydrophobic silicon substrates, segments of adsorbed particles that consists of both mono- and multilayers were observed (Figure 8a); which otherwise seem to occupy a smaller surface area compared to the highly hydrophilic substrates. In contrast, the latter have a larger particles distribution area (Figure 8b). This primarily results from high surface energy of hydrophilic surfaces which consequently attracts the water (i.e.., particles carrier), thereby facilitating surface wetting. In Figure 8c, which is a magnified view of Figure 8b, we show a segment of monolayer particles arrangements arising from tip-substrate contact on a hydrophilic surface. It should be noted that the multi-layer cluster, observed on the hydrophobic surface (Figure 8a), is a consequence of the water-repulsive nature of these surfaces, which clearly limits their wettability. It is therefore evident that the particle-imprint approach can favorably work well on hydrophilic surfaces to realize monolayer particle assembly which facilitates and guarantees accurate particle counting/estimation. 

## 4. Cantilever-Based Particle Mass Detection

### 4.1. Cantilever Sensor Cleaning/Preparartion 

As a pre-requisite, the cantilever sensors were cleaned to guarantee a contaminants-free surface for accurate determination of the initial (bare) cantilever mass *m*_0_ and resonant frequency *f*_0_. This consequently enhances the accuracy of determining the adsorbed particles mass, Δ*m*. The actual cantilever mass *m*_0_ and corresponding standard uncertainty for the fabricated sensors ranged from 4.26 µg ± 0.10 µg, 15.01 µg ± 0.10 µg to 23.01 µg ± 0.19 µg for TCant2, RCant1, and TCant1 cantilever types, respectively. Moreover, their typical mass sensitivities *S*_m_ were approximately 0.13 Hz/pg (TCant2), 0.03 Hz/pg (RCant1), and 0.02 Hz/pg (TCant1). The parameter $S_{m}\;\approx \;\frac{f_{0}}{2m_{\mathrm{eff}}}$, in which *m*_eff_ denotes the effective mass of the cantilever; and *m*_eff_ is about ¼ of the static mass of a bare cantilever [35]. The surfaces of our cantilevers are not functionalized but contain a native oxide which makes them hydrophilic and suitable for particle adsorption.

Due to the strong van der Waals forces between magnetic polystyrene (MPS) microparticles and the (hydrophilic) silicon surface, the sticky magnetic polystyrene particles would not ordinarily be desorbed by soaking in acetone but through a controlled sonication process. It should however be noted that removing of MPS μPs from silicon substrate surfaces is nevertheless possible through exclusive wet cleaning processes. These processes primarily involve the use of alkaline and acidic solutions. For instance, the particle removal efficiency using alkaline solutions was demonstrated by Itano et al. to be superior to acid solutions [36]. However, these alkaline solutions (e.g., NH_4_OH-H_2_O_2_-H_2_O) etch the silicon surface (by ~0.25 nm/min or more) to lift off the particles, and then dislodge them from the silicon surface by electrical repulsion. On the other hand, acidic solutions such as H_2_SO_4_-H_2_O_2_ solution oxidize absorbed particles and decompose them by the strong oxidizing force [36]. Nonetheless, both alkaline and acidic solutions are etch-based particle removal techniques with a high risk of damaging the cantilever features e.g., the piezoresistive Wheatstone bridge and the electrical connection lines.

Contrarily, ultrasonic cleaning technology utilizes ultrasonic energy to agitate the particles (or contaminants) on the solid surface and a liquid (solvent) to rinse the loosened particles away. This method can most conveniently be used to remove contaminants in hard-to-reach areas. Moreover, the process takes considerably shorter cleaning time compared to the normal wet cleaning processes. Furthermore, it is a thorough process which yields high-quality cleaning. Nevertheless, the use of high intensity of vibrations (in which cantilever sensor is subjected to during cleaning) can potentially damage or destroy the sensor.

**Figure 9:**
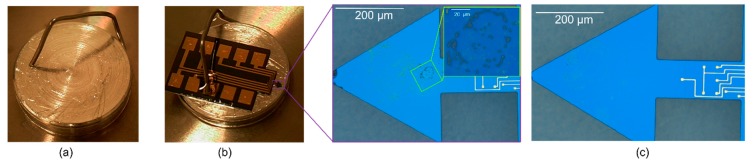
Optical images of (**a**) a specially designed metallic adaptor, (**b**) TCant1 cantilever (adsorbed with magnetic polystyrene particles) mounted and clipped on a metallic adaptor (prior to immersing it in acetone for sonication). The surface of the cantilever after sonication cleaning is shown in (**c**).

To mitigate this challenge, we devised and assembled a metallic adaptor (shown Figure 9a) onto which the cantilever sensor (adsorbed with MPS µPs) was mounted and clipped (Figure 9b). Such clipping was necessary to ensure that the sensor does not randomly vibrate and move with the liquid and accidently hit the wall of the glass beaker. The whole assembly was then immersed into a glass containing acetone for sonication (which lasted barely less than 3 min). After cleaning, optical inspection (Figure 9c) was done to assess the effectiveness of the particle removal process. Furthermore, the cantilever resonant frequency was again measured to ascertain, compare, and average with the pre-desorption resonant frequency value.

### 4.2. Resonant-Based Mass Measurement

#### 4.2.1. Gravimetric Mass Sensing

**Figure 10:**
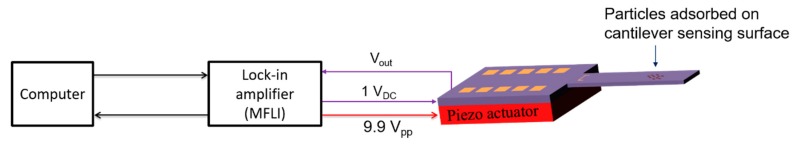
A schematic setup for the measurement of the cantilever resonant frequency *f*_0_ responses to determine the mass Δ*m* of the adsorbed particles. The lock-in-amplifier (MFLI) supplies voltage to the in-plane piezo actuator and Wheatstone bridge of the piezoresistive silicon cantilever sensor.

In determining the mass of the adsorbate, a gravimetric measurement setup was deployed as schematically shown in Figure 10. The cantilever was excited in its fundamental in-plane mode using a piezo actuator (P-121.01, from PI Ceramics GmbH, Lederhose, Germany), while mechanical vibrations thereto were detected piezoresistively by means of a U-shaped Wheatstone bridge embedded in the sensor during the fabrication process. The direct current (DC) voltage to the Wheatstone bridge (1 V) and sinusoidal actuation signal (up-to 9.9 V_pk_) to the piezo actuator were supplied by a lock-in-amplifier instrument (MFLI, Zurich Instruments Ltd, Zurich, Switzerland). All connections to and from the MFLI instrument to the piezo actuator and cantilever were accomplished via SMA connectors and coaxial cables.

Initially, the resonant frequency *f*_0_ of a pre-cleaned bare cantilever sensor was measured. Upon depositing, and after vaporizing the particle solution from the sensing surface, the particle-induced fundamental resonant frequency *f*_0_^′^ was then measured under ambient conditions. In this case, a shift in resonant frequency between the bare and the mass-loaded cantilever was obtained:

(1) $$\Delta f=f_{0}^{\prime }-f_{0}.$$

**Figure 11:**
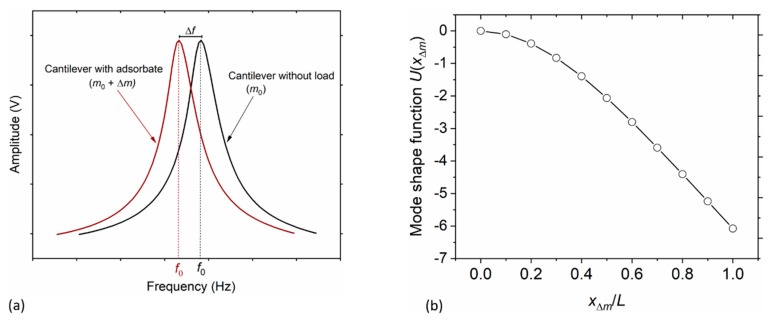
(**a**) Schematic representation of the shift Δ*f* in resonant frequency of a cantilever with/without an adsorbate (load, Δ*m*). (**b**) Mode shape function (calculated based on Equation (3)) at a point *x*_Δ*m*_/*L* along the cantilever beam of length *L* vibrating in the first (fundamental) mode of which *λ*≈ 1.8751. In our experimental work, typical particle adsorption positions ranged from *x*_Δ*m*_/*L* ≈ 0.4 to 0.8.

This frequency shift Δ*f* (depicted in Figure 11a) is linked to the adsorbate mass (Δ*m*) in accordance with Equation (2). Given the knowledge of the adsorption position *x* (i.e., the distance between the loaded mass and the fixed end of the microcantilever with beam length *L*) and cantilever mass *m*_0_, the value of Δ*m* can most conveniently be calculated using [37]:

(2) $$\Delta m=-\frac{2m_{0}}{U^{2}{(x}_{\Delta m})}\frac{\Delta f}{f_{0}},$$

where,

(3) $$U\left(x_{\Delta m}\right)=\left(\cos\;\lambda +\cos h\;\lambda \right)\left(\cos(\lambda \frac{x}{L})-\cos h(\lambda \frac{x}{L})\right)+\left(\sin\lambda -\;\sin h\;\lambda \right)\left(\sin(\lambda \frac{x}{L})-\sin h\left(\lambda \frac{x}{L}\right)\right),$$

is the mode shape function of the cantilever, in which *λ* denotes the vibration modal constant. The value of *λ* is simply the product of the modal wavenumber *β*_n_ and the length *L* of the cantilever beam, i.e., $\lambda =\beta_{n}L$; and it depends on the vibrational mode number (which is an integer number n ≥ 1). In our dynamic frequency response measurements, only the fundamental flexural mode (i.e., first vibrational mode, n = 1) was mainly involved; hence, *λ* ≈ 1.8751 [35]. The calculated fundamental mode shape function in relation to the (normalized) particle adsorption point *x*_Δ*m*_/*L* is shown in Figure 11.

In determining the position *x*_Δ*m*_, SEM images were captured for each successfully prepared cantilever sample and *x*_Δ*m*_ was estimated using ImageJ [38]. The position of the adsorbed magnetic particles was determined from SEM images with an accuracy of approximately ± 0.01 µm. The SEM particles imaging was mainly done after measuring the resonant frequencies of each of the involved samples. 

Nonetheless, if we assume a distributed mass condition of the adsorbed particles, then:

(4) $$\Delta m=2m_{\mathrm{eff}}\frac{\Delta f}{f_{0}},$$

where, *m*_eff_ ≈ *m*_0_/4 denotes the effective mass of the cantilever sensor.

#### 4.2.2. Particle Mass Determination

The frequency shift Δ*f* (determined in accordance with Equation (1)) was used to compute the particle mass Δ*m* using Equations (2) and (3) or Equation (4). This was done by averaging at least five frequency sweeps (both before and upon particle adsorption on the sensor, and after particle removal i.e., cleaning of the sensor). 

**Figure 12:**
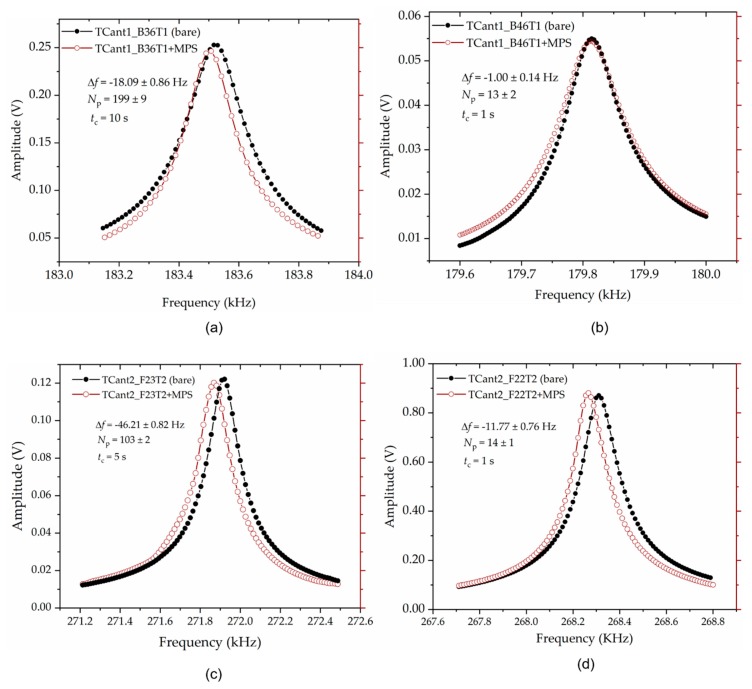
Plot of the resonant frequency responses before and after adsorbing magnetic polystyrene (MPS) microparticles on assorted fabricated triangular cantilevers. (**a**,**b**) denote measurements from TCant1 cantilever sensors, while (**c**,**d**) are measurements from TCant2 cantilevers. The MPS µPs (whose number *N*_p_—determined based on point mass-condition i.e., from Equations (2) and (3)) were deposited on the sensing area through the particle-imprint approach by observing tip-sensor-contact time *t*_c_ of about 10 s, 5 s, and 1 s using stainless steel tips with nominal internal diameters of ~0.10 mm (**a**,**c**,**d**) and ~0.15 mm (**b**).

Typical resonance frequency *f*_0_ responses of a cantilever with and without adsorbed magnetic polystyrene particles are, respectively, depicted by the brown/open and black/full lines/circles in Figure 12. The particles-induced resonant-frequency shift Δ*f* = −18.09 Hz ± 0.86 Hz and −1.00 Hz ± 0.14 Hz (delineated in Figure 12a,b) corresponds to measurements from TCant1 sensors with *f*_0_ ~ 183.5 kHz and 179.8 kHz, respectively; while, Δ*f* = −38.91 Hz ± 0.61 Hz and −11.77 Hz ± 0.76 Hz (shown in Figure 12c,d) resulted from TCant2 sensors with *f*_0_ ~ 271.9 kHz and 268.3 kHz, respectively. These Δ*f* values together with their uncertainties were computed from repeated frequency response measurements. The initial resonant frequency of the fabricated cantilevers of the same category (e.g., TCant2 sensors with *f*_0_ = 271.9 kHz and 268.3 kHz as depicted in Figure 12c,d) are slightly different, supposedly due to small variations in the sensor dimensions arising from the fabrication process. Primarily, for the in-plane excited cantilever sensors, *f*_0_ ∝ *w*/*L*^2^. Typically, small changes in the beam width *w* and/or length *L* of the cantilever may emanate from the resolution of our photolithography (~1 µm). Correspondingly, therefore, this affects the expected resonant frequency *f*_0_ by approximately ±1.2 kHz, for triangular cantilever sensors. On the other hand, variations in cantilever thickness *t* may also result mainly from the membrane etching process. But this affects the effective cantilever mass (and its sensitivity *S*_m_).

If we consider point-mass condition (Equations (2) and (3)) and thereby compute the particles mass Δ*m*, the resonant frequency shifts (delineated in Figure 12a–d), translate to the particles concentration *N*_p_ of about 199 ± 9 and 13 ± 2, from TCant1 sensors; and 103 ± 2 and 14 ± 1, from TCant2 sensor. This clearly shows that the number of particles, for each sensor category, increased with resonant frequency shift. The values of *N*_p_ were determined from the mass ratios of Δ*m* to a single magnetic polystyrene particle mass, i.e., 3.53 pg ± 0.25pg determined from its measured volume (diameter) and given density. Besides MPS particles, PMMA µPs were similarly imprinted on the cantilever sensor (*f*_0_ ≈ 181.0 kHz) and yielded *N*_p_ ≈ 35, resulting from a resonance shift Δ*f* ~ −9.88 Hz. To calculate the mass Δ*m* using Equations (2) and (3), the position of the adsorbate (i.e., *x*_Δ*m*_ = 470 µm to 750 µm) on the sensor was measured from SEM images (using ImageJ as earlier discussed in Section 4.2.1). 

It is worth noting that the vibration amplitudes of the cantilever sensors with and without load (as depicted in Figure 12) were closely in agreement, with a small difference of less than 2%. This may connotate a small change in mechanical quality factor (i.e., damping); but it does not however affect the cantilever resonant frequency and the shift thereto nor the adsorbate mass (computed therefrom). Nevertheless, the measured *Q* values for TCant2 sensors (→ Figure 12c,d) were relatively higher (*Q* ~ 3000 ± 150, which depicts better stability) compared to TCant1 cantilevers (→ Figure 12a,b) with *Q* ~ 1800 ± 200. Furthermore, as expected—in accordance with Equation (2), different cantilever frequency bands were observed (in Figure 12a–d). This was typically due to the differences in the number of adsorbed particles (*N*_p_ or Δ*m*) on each sensor, small variations in adsorbate position (i.e., *x*_Δ*m*_), and cantilever mass sensitivity (*S*_m_).

**Figure 13:**
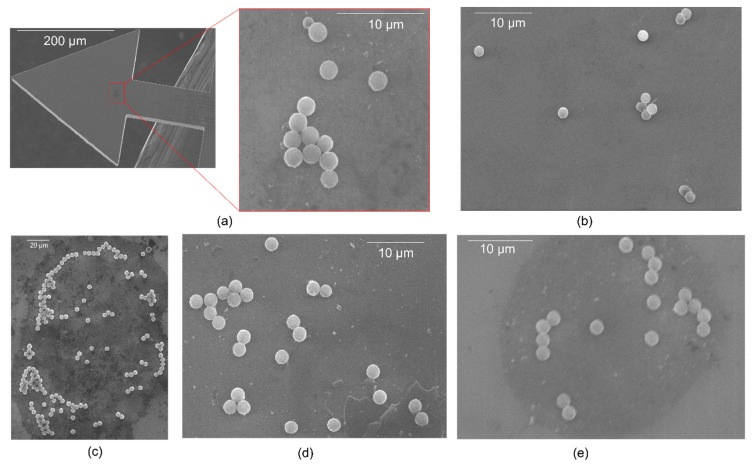
Typical SEM images of the adsorbates of magnetic polystyrene (MPS) microparticles on our various silicon-based piezoresistive TCant1 and TCant2 triangular cantilever sensors with (**a**–**e**) respectively denoting B46T1, F22T2, B24T1, A16T1, and A14T1 cantilevers. The SEM-counted number of particles in (**a**–**e**) are ~13, 11, 160, 25, and 18, respectively. From all the cantilever samples, a monolayer particle assembly was clearly observed. These MPS µPs were deposited on the cantilevers utilizing the particle-imprint method.

After resonant frequency measurements, the particles-imprinted cantilever samples were microscopically analyzed (using SEM) to examine the nature of particle arrangement on the sensing surface. In all these cases, for instance, as depicted in Figure 13, the adsorbed particles resulted in monolayer particles assembly. Consequently, for the assorted SEM images delineated in Figure 13a–e, a particles-count of about 13, 11, 160, 25, and 18 was determined, respectively. In Figure 13a, for instance, an SEM-particles count of about 13 magnetic polystyrene particles was obtained (on TCant1 sensor) i.e., 12 equally sized (~1.83 µm ± 0.03 µm) and 1 oddly sized (~1.07 µm ± 0.03 µm) particles. Considering the density and volume of the particles, this SEM-particle count translates to a calculated mass of about 43.06 pg ± 0.16 pg, which agrees well with the particles mass Δ*m* ≈ 44.39 pg ± 6.07 pg (i.e., *N*_p_ ≈ 13 ± 2) determined from the corresponding resonant-frequency response (delineated in Figure 12b). A small difference between the two mass estimates was nevertheless observed. Besides, the frequency shift (Δ*f* ≈ −1.00 Hz) was notably small. Consequently, this necessitated an enhanced mass sensitivity of our sensors. Considerably, this limit was fairly extended based on our TCant2 cantilever design; from which, we realized a mass sensitivity *S_m_* ~0.13 Hz/pg, and gravimetrically detected (Figure 12d) and determined (based on point mass-condition) about 14 MPS µPs, with a better frequency resolution. This number of MPS particles (i.e., *N*_p_ ≈ 14) is similarly in good agreement with the corresponding observed particles-count from the SEM image (in Figure 13b), i.e., *N*_p_ = 11. Furthermore, in our recent works [39], miniaturized sensors for an enhanced airborne particles detectability has been demonstrated (with *m*_0_ = 2 ng to 5 ng and a mass sensitivity ~0.13 Hz/fg); and their use in liquid-based particles detection is further intended. Besides, special consideration is intended to apply our particle sampling and imprinting approach on commercial piezoresistive silicon cantilever sensors i.e., CAN30-1-2 sensor, from CiS Forschungsinstitut für Mikrosensorik GmbH, with *m*_0_ ≈ 20 µg; and, PRSA-L300-F80-TL sensor, from SCL-Sensor. Tech. Fabrication GmbH, with *m*_0_~0.5 µg (a factor of 30 to 50 lower than our in-house fabricated cantilevers (Table 1)).

**Figure 14:**
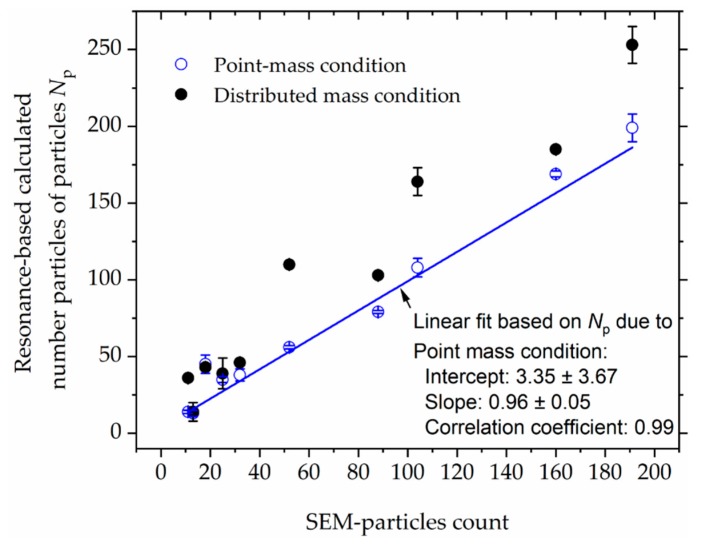
Comparison of the number of magnetic polystyrene microparticles on both TCant1 and TCant2 cantilever sensors. The number of particles *N*_p_ was determined from the measured resonant frequency responses (considering both point-mass and distributed mass conditions) and is compared with the SEM-particle counting. The blue line depicts an error-weighted linear fit for *N*_p_ at point-mass condition.

In Figure 14, we additionally show a correlation plot that compares the SEM particle-analysis results with the calculated number of particles from resonant-frequency responses (considering both point-mass and distributed-mass conditions). The latter condition assumes that the mass of the adsorbate is evenly distributed on the sensing surface; a factor that potentially leads to the poor correlation between the calculated number of particles (due to distributed mass condition) and the observed (SEM-) particle counts, as depicted in Figure 14. On the other hand, from the same plot, it is apparently clear that the resonance-based number of particles due to point-mass condition (cf. Equations (2) and (3)) is highly correlated with the (SEM)-particle counts. With a correlation coefficient of nearly 0.99 (→ error-weighted linear fitting in Figure 14) and a minimum detection limit of about 0.05pg (exhibited from TCant2 sensors), it shows that the measurements are pretty much in agreement and our (TCant2) sensors offer reasonably high sensitivity, respectively. Notably though, some deviations from ideal correlation of the resonance-based and SEM-particle estimations were observed (Figure 14) and their possible causes will further be considered in the subsequent section, in which we will discuss an exemplar of assorted possible measurement influences.

Nonetheless, considering the small amount of liquid realizable through the on-cantilever particle imprinting process coupled with the depicted high sensitivities, it is worth noting that the measurement approach presented herein can plausibly be applied in the testing or detection of liquid-borne viruses (e.g., coronavirus—whose primary mode of transmission from person to person is through virus-laden droplets). In testing for coronavirus, for instance, a test fluid e.g., sputum (from a suspected or infected person) is sampled. Apparently, most coronavirus rapid-testing methods detect the antibodies produced in response to viral infection [40]. Contrastingly, to detect the presence of coronavirus itself, a MEMS-cantilever-based sensor is desirable. This has recently been demonstrated by Digital Diagnostics AG by functionalizing the surface of the cantilever sensor with a capture layer of antibodies, which binds antibodies in a test sample fluid [41]. Given the flexibility and ease of adaptation of the on-cantilever particle imprinting approach, we believe it can potentially and cost-effectively be utilized to detect liquid-borne viruses such as coronavirus. 

### 4.3. Assessment of Measurement Uncertainty

**Figure 15:**
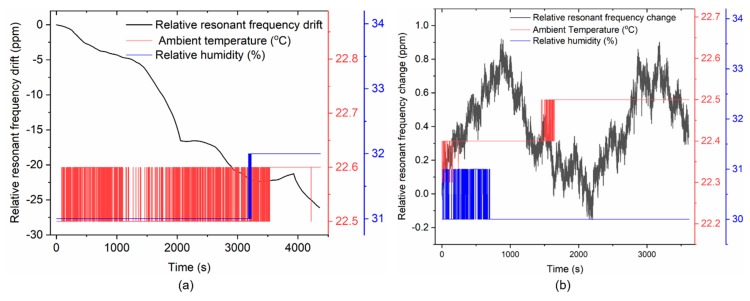
Relative resonant frequency drift (black line) of bare, silicon triangular cantilevers (**a**) TCant1 (*f*_0_ ≈ 181.265 kHz) and (**b**) TCant2 (*f*_0_ ≈ 262.253 kHz) under typical ambient temperature (red line) and relative humidity (blue line) conditions in the laboratory over measurement time (~1 h).

Resonant-based mass sensing, like conventional macroscale mass measurements [42], is influenced by the loading position and environmental conditions such as relative humidity (*RH*) and temperature (*T*). A temperature change (Δ*T*) correspondingly changes *f*_0_ by multiplying it with $\frac{\left(\alpha +\alpha_{E}\right)}{2}\Delta T$ [35], where *α* = 2.6 ppm/K and *α_E_* = −44 ppm/K [35,43] denote the linear coefficient of thermal expansion and temperature coefficient of Young’s modulus *E* of silicon, respectively; while the change in relative humidity (Δ*RH*) is bound to decrease *f*_0_ due to the moisture that cling to the sensor surface. In our measurement process, these variables were monitored for every measurement cycle and found to be small. For instance, the observed maximum temperature change Δ*T* for TCant1 sensor—delineated in Figure 15a—was ~0.1 °C within one hour, while the change in relative humidity Δ*RH* during the same period was ~1%. Similarly, for TCant2 sensor (Figure 15b), Δ*T* ≈ 0.2 °C, and Δ*RH* *≈* 1%. 

Furthermore, we define the relative resonant frequency drift *y*(*t*) (shown in Figure 15) as a ratio of the measured resonance frequency *f*_0_(*t*), minus its initial value at *t* = *t*_0_, and *f*_0_ (*t*_0_), i.e.,:

(5) $$y\left(t\right)=\frac{\Delta f}{f_{0}}=\frac{f_{0}\left(t\right)-f_{0}\left(t_{0}\right)}{f_{0}\left(t_{0}\right)},$$

which yields an overall relative frequency drift of about −26.1 ppm and 0.9 ppm for TCant1 and TCant2 sensors, respectively. It should however be noted that the typical duration for measuring the resonance frequency responses for each cantilever sensor (with/without magnetic particles) was about 1 to 5 min. Within this short measuring period (i.e., within 5 min), a maximum relatively relative frequency drift of 1.2 ppm (TCant1 sensor) and 0.4 ppm (TCant2 sensor) was observed, i.e., more than an order of magnitude lower than the overall (~1 h) observed drift values of ~−26.1ppm (Figure 15a) and ~0.9 ppm (Figure 15b), respectively. Furthermore, the effect of humidity can be assumed to be negligible (yielding less than 1ppm for Δ*RH* ≈ 1% [44]).

Further measurement influences (based on Equation (2)) included uncertainties associated with the determination of the mass of the cantilever sensor (primarily from the thickness of the sensor), particle(s) adsorption position *x*_Δ*m*_ along the cantilever beam (mainly due to tip alignment; of which, we consider the resolution of the positioning system) and the repeatability of resonant-frequency shift. Moreover, for the calculated number of particles (cf. Figure 14), particle-diameter estimation was a critical-influencing parameter. All particles were essentially assumed to be identical in shape (spherical) and size (~2 µm). This was typically observed in all the analyzed samples except one (Figure 13a) in which only one oddly sized particle (~1 µm) was observed. Besides, to minimize particle mass measurement uncertainties, further work involving calibration of the in-plane cantilever stiffness is intended.

## 5. Conclusions

In this study, we have designed and fabricated a triangular cantilever and utilized it as a sensing surface upon which a novel particle-imprinting approach has been implemented. With this approach, liquid-borne particles were sampled by dipping a dispensing tip into an arbitrary-sized particle-laden droplet (on a hydrophobic silicon substrate) followed by tip-sensor contact. We have also examined a few crucial parameters that regulated the number of particles adsorbed and assembled on the silicon sensors/substrates i.e., surface wettability, tip size, and contact time. With the particle-imprint approach, herein presented, the need for a dispensing-air pressure (essentially utilized in liquid dispensing) was eliminated. Furthermore, no complicated instrumentation is required to deposit numerable particles; hence, making it fairly cost-effective. Using on-cantilever imprinting method, a monolayer-particle assembly has been realized on hydrophilic silicon cantilever sensors with the lowest particles-count of about 11. Typically, from our fabricated triangular microcantilever mass sensors (i.e., TCant1 and TCant2), we can realize a minimum detectable frequency shift Δ*f*_min_ of about 8 mHz and 7 mHz, respectively. We have also realized a higher resonant frequency of the TCant2 sensors, which was 271 kHz compared to a rectangular (regular) cantilever sensor (220 kHz) of equivalent total length (*L* = 1 mm) and mass (*m* ≈ 5.94 µg). This effectively offered the TCant2 sensor superior advantages (over RCant1) with a higher mechanical quality factor (*Q* ~ 3000), an enhanced mass sensitivity (*S*_m_ ≈ 0.13 Hz/pg), and a lower minimum detectable mass (~Δ*f*_min_/*S*_m_ ≈ 0.05 pg). In this regard, therefore, we have further envisaged the possibility of utilizing the on-cantilever imprint approach to measure/detect liquid-borne viruses (e.g., coronavirus) and single particles using our sensors. We have also briefly examined main sources of uncertainty which affected our particle sampling and resonant frequency response measurements.

## Figures and Tables

**Table 1:** Cantilevers design parameters and simulated characteristics.

Symbol	Definition	Cantilever Sensor Design/Type		
		Rectangular (RCant1)	Triangular (TCant1)	Triangular (TCant2)
*L*_1_	Rectangular step-length (µm)	-	394	394
*L*_2_	Triangular step-length (µm)	-	606	606
*L*	Cantilever total length (µm)	1000	1000	1000
*w*	Width (fixed-end) (µm)	170	170	170
*t*	Thickness (µm)	15	15	15
*b*	Triangular-free-end: base (µm)	-	700	340
*m*_0_	Cantilever mass (µg)	5.94	9.76	5.94
*f*_0_	Resonant frequency (kHz)	220	185	271
*S_m_*	Mass sensitivity (Hz/pg)	0.07	0.04	0.09
*Q*	Quality factor ^(i)^	2000 ± 200	1800 ± 200	3000 ± 150

(i) Measured mechanical quality factor.
